# Beyond regulatory compression: confronting the liminal spaces of health research regulation

**DOI:** 10.1080/17579961.2016.1250378

**Published:** 2016-12-01

**Authors:** Samuel Taylor-Alexander, Edward S. Dove, Isabel Fletcher, Agomoni Ganguli Mitra, Catriona McMillan, Graeme Laurie

**Affiliations:** ^a^ J Kenyon Mason Institute for Medicine, Life Sciences and the Law, School of Law, University of Edinburgh, Edinburgh, UK

**Keywords:** Health research, law, liminality, process, regulation

## Abstract

Biomedicine and the life sciences continuously rearrange the relationship between culture and biology. In consequence, we increasingly look for a suitable regulatory response to reduce perceived uncertainty and instability. This article examines the full implications of this ‘regulatory turn’ by drawing on the anthropological concept of liminality. We offer the term ‘regulatory compression’ to characterise the effects of extant regulatory approaches on health research practices. With its focus on transformation and the ‘in-between’, liminality allows us to see how regulatory frameworks rely on a silo-based approach to classifying and regulating research objects such that they: (1) limit the flexibility necessary in clinical and laboratory research; (2) result in the emergence of unregulated spaces that lie between the bounded regulatory spheres; and (3) curtail modes of public participation in the health research enterprise. We suggest there is a need to develop the notion of ‘processual regulation’, a novel framework that requires a temporal-spatial examination of regulatory spaces and practices as these are experienced by all actors, including the relationship of actors with the objects of regulation.

## 1. Introduction

Biomedicine and the life sciences continuously rearrange the relationship between culture and biology. At times, the fields can both problematise what it means to be human (consider, for example, xenotransplantation) and also create uncertainty and instability in individual and public life (consider, for example, the human tissue retention ‘scandal’ in the UK in the late 1990s). When this occurs, we often look for a suitable regulatory response to reduce the uncertainty and instability by mitigating potential risks and harms and by directing or influencing actors’ behaviour to accord with socially accepted norms and/or to promote desirable social outcomes. In this article, we examine the full implications of this ‘regulatory turn’ – and in particular the command-and-control model within the broad gamut of regulatory paradigms that exists – by drawing on the anthropological concept of liminality. Developed to make sense of ritual, structure, and agency, the notion of liminality refers to a threshold phase characterised by uncertainty, possibility, marginality, and transformation. We argue that liminality can yield novel insights into the nature of health research regulation, namely by (1) helping us to better understand the profound sociotechnical challenges that continue to redefine life, and (2) exploring alternative paths to governing the behaviour of actors and enforcing norms across sites of authority in health research. In doing so, we offer the notion of ‘regulatory compression’ to characterise the effects of extant regulatory approaches on health research practices.

This article is divided into four sections. The first section claims that the current mainstream apparatus of regulation fails to address adequately the full array of socio-technical concerns in health research. Using the frame of command-and-control regulation as a particularly acute instance of regulation given effect through law, we posit that such responses often compress and dislocate the ‘feedback loops’ needed for robust and dynamic steering of behaviour, thus stunting the development of flexible regulatory tools that can better address health research. The second section focuses on the evolution of liminality, from its original use to analyse ‘rites of passage’ to its development as a conceptual tool for characterising and understanding contemporary practices. In drawing attention to questions of process, ‘anti-structure’ and other ontologies, scholars have exposed the disruptive potential of liminality: the ‘noise’ generated in and across liminal spaces requires various actors to systematically transform these putative interferences into new forms of ‘inter-reference’ – that is, forms of understanding that incorporate and go beyond disciplined ways of knowing the world. In the last two sections, we reveal the potential significance of a liminal approach to health research regulation by envisioning its application in areas such as research ethics review and human embryo research as well as its application to other kinds of actors, including things. We also investigate the space-time metaphor of liminality and the notion of ‘social value’ in research. Finally, we conclude with broader thoughts about how liminality enables us to reimagine and reconceptualise the nature of health research regulation.

## 2. Regulatory compression

The metaphor of the ‘regulatory space’ is now well entrenched in academic and everyday parlance,^1^ its modern origins being attributed to the seminal work of Hancher and Moran, who argued that in order to understand regulation, attention must be paid to the physical places where regulation occurs.^2^ Morgan and Yeung have further suggested that two important implications arise from this, namely, that (1) there is limited relevance of law and formal authority within such spaces, and (2) multiple dynamics, including history, culture, and organisational arrangements, will impact significantly on regulatory dynamics.^3^ In terms of regulatory theory, multiple approaches to creating regulatory spaces can be adopted, including rules-based regulation,^4^ principles-based regulation,^5^ risk-based regulation,^6^ and archetypically, command-and-control regulation which sits at the extreme end of the regulatory spectrum and is typified by the top-down imposition of standards of conduct supported by the threat of criminal sanction.^7^ Thus, while being something of a caricature of a regulatory approach, this last example can nonetheless be illustrative of common dynamics that occur in regulatory space. As such, it serves as a useful exemplar for the present discussion.

The main contention of this article is that liminality occurs in multiple regulatory spaces and, moreover, that by recognising and acknowledging this we can encourage a radical reimagining of those spaces by revealing important processes that remain outside the purview of the traditional regulatory responses. To consider command-and-control in particular, such an approach is characterised by what we call *regulatory compression*. While feedback loops – outputs of a regulatory system routed back as inputs to the various actors implicated in the enterprise – exist between research and regulatory spaces,^8^ they are bound by the organisational structures in which they arise. The temporal dimensions of health research regulation play a central role in mediating the resolution of ontological issues (of what something ‘is’ that is to be regulated) and of democracy (how can *we* decide appropriate and socially acceptable ways of regulating). When the regulatory space is viewed this way, we can see the effects of respective regulatory approaches in health research practices. For example, the materiality of regulatory objects, such as a face for transplantation or an embryo in the laboratory, changes throughout a given research protocol or experimental procedure. However, regulation often fails to recognise the fluid nature of the objects that it regulates, frequently forcing researchers to adapt their practices (and the research objects themselves) to bring them into line with norms and guidelines.

More acutely, in circumstances of genuinely disruptive change brought on by rapid advances in technological development, and when this change threatens established behaviours and working practices, such as smart devices or autonomous vehicles, regulation can often be called upon to fill new regulatory ‘gaps’ in a form of rapid response mode. Too often, however, this can result in ill-conceived and foreshortened regulation that is scarcely fit for purpose.^9^ Thus, regulatory compression speaks to the tendency for regulation to promote a rigid, fractured, and sometimes top-down response to ethical, epistemic, and ontological issues and to undervalue temporal and democratic values. We see this, for example, in the demarcation of what counts as ‘tissue’, ‘organ’ or ‘data’ to accord with, e.g. the Human Tissue Act 2004 or Data Protection Act 1998 (not to mention with institutional regimes created to regulate these ‘bounded objects’), even though everyday scientific practice suggests there are many blurred boundaries between such materials. Thus, research is done with tissue to produce data that reveal information that is interpreted to reveal novel insights that lead to new knowledge about the world.

The language and reasoning central to the work of command-and-control health research regulation has been criticised on two grounds. First, it fails to achieve the appropriate distance from the objects of its analysis – a problem of either regulatory capture or regulatory naïveté. For example, bioethics is a critical component – a social movement and professional discipline – of modern research regulation. Regulations around the world have sprung out of the work derived from, inter alia, the World Medical Association and the Council for International Organisations of Medical Sciences.^10^ Yet bioethics per se – a field that regulators rely on as an intermediary between research and policymaking settings – is often divorced from the experiences of patients and practitioners^11^ and too closely aligned to the practices that it seeks to govern.^12^ Rather than being able to capture adequately the complexities of medical research, research participant experiences, and clinical life, bioethics policy has been shown to play an integral part in the production of this complexity and the shaping of corresponding responses to the scientific and moral uncertainty that accompanies it.^13^ A central feature of these responses is the production of new regulations that often contrast directly with the flexibility and fluidity at play in the biosciences.^14^

The second critique of analytical distance suggests that questionable research practices are institutionally mandated because regulatory reasoning only allows certain questions to be asked.^15^ Alongside this are important issues such as: the rights of individuals and communities that live in areas with limited regulatory recourse, the nature and scope of the forms of protection they should receive, and how this protection can be fostered. As Kelleher writes, despite the existence of a host of guidelines for conducting offshore clinical trials, for example, there is a dearth of on-the-ground mechanisms for protecting trial communities.^16^ Adding to these difficulties are the modes of ‘strategic ignorance’^17^ that are tactically employed in research contexts to produce authority in the face of unsettling information that could stymie scientific progress or result in liability or guilt in the aftermath of deleterious events. Consider, for example, the fallout following the Food and Drug Administration’s licensing of Ketek, an antibiotic pharmaceutical that was subsequently linked to liver failure. Analysis of the enquiry suggests ‘that in drug regulation, different actors, from physicians to regulators to manufacturers, often battle over who can attest to the *least* knowledge of the efficacy and safety of different drugs’.^18^

In sum, current command-and-control approaches to novel medical technologies have been criticised as undemocratic,^19^ temporally limited,^20^ unwaveringly supportive rather than critical of technological and scientific trajectories,^21^ and generally burdensome and costly.^22^ We see at least three core effects, then, of regulatory compression: (1) a limiting of the flexibility (or indeed serendipity) intrinsic to health research, which could both unduly hinder research and disregard the interest of research participants; (2) a marginalisation of publics’ participation in health research projects or disregard of their concerns; and (3) the emergence of unregulated spaces that lie between the bounded regulatory spheres.

Building on the above critiques, a number of scholars have begun to outline new approaches for engaging with health research regulation. As well as refiguring the temporal dimensions of oversight as a process that must be examined over its life course, these scholars stress the need to rethink the relation between the different actors involved in research practices. Anthropologists Paul Rabinow and Gaymon Bennet, for example, speak of ‘designing human practices’ as an iterative and synergistic process aligning actors from different disciplines in order to build responses to challenges from the outset of research.^23^ Jane Kaye and colleagues write of ‘ELSI 2.0’ as a ‘collaborative’ enterprise with an epistemic and institutional architecture that fosters novel forms of coordination and enables adequate responses to the profound changes wrought by contemporary health research.^24^ However, largely absent from these proposed models is substantive discussion of engaging either with the diverse publics that unite around research, or with society in general. The benefits of such engagement have been outlined elsewhere, and developed through such notions as ‘partnership’^25^ and ‘solidarity’.^26^ While these latter works speak directly to the democratic limits of any current command-and-control approach, we argue here that a processual approach to health research, grounded in liminality, suggests they need to be coupled with models that promote iterative and flexible regulation and critiques of the broader political purpose implicit in research regulation.

## 3. Liminality: its development and its potential

The concept of liminality offers a unique purview to (re)consider extant approaches to health research regulation. In this section, we stress the need to move away from a tendency of some of the liminality literature to employ the word as a synonym for something or someone that occupies a ‘marginal’ space. When viewed in the context of health research regulation, these liminal spaces do *not* occupy the periphery. On the contrary, our analysis shows that liminality is *central* to the everyday research practices and the regulatory mechanisms that surround them.

Liminality challenges us to engage with the *processual and experiential* dynamics of research, including the ways in which practices, people, and entities are affected by regulation. This focus on process and experience emerged originally in the French anthropologist Arnold Van Gennep’s ethnographic research of ritual practices.^27^ Seeking to understand social transformation, Van Gennep identified ‘liminal rites’ as a critical component of the reproduction of social order, positing a tripartite model that outlined: (1) the symbolic and spatial separation of an individual from their existing social position (pre-liminal); (2) the transformation of their social status as they pass through an adjacent, often marginal space characterised by a dissolution of established social order and hierarchy (liminal); and (3) their spatial and symbolic reincorporation into society (post-liminal). Because the suspension of social order is spatially and temporally limited, such ritual practice allows for social transformation to occur in a manner that preserves broader organisational structures.

Building on this interpretation of ritual practices, in the mid-twentieth century, the British-born anthropologist Victor Turner moved attention to liminal experiences in non-ritual societies. His work suggests that it is through liminality that we see and experience the most basic elements of common humanity. In his seminal work, Turner examined the role of liminality in ritual performances, such as initiation rites, describing the transformative dimensions of liminal experience while noting that such performances are often evocative and revealing of a society’s underlying structure and values.^28^ According to Turner: ‘Liminal entities are neither here nor there; they are betwixt and between the positions assigned and arrayed by law, custom, convention, and ceremony’;^29^ liminality ‘may partly be described as a stage of reflection’.^30^ For Turner, liminality is an ‘inter-structural situation’ that involves a coincidence of opposite symbols: ‘This coincidence of opposite processes and notions in a single representative characterizes the peculiar unity of the liminal: that which is neither this nor that, and yet is both.’^31^ Because it accompanies the dissolution of social order and established hierarchies, the liminal time-space can be regarded as a nexus for transformation and reflection. That is, engaging with process and change helps reveal existing social structures and ordering practices. Moreover, as Thomassen writes, while liminality is a central component of modernity it should be used to examine, rather than to explain, social phenomena. In doing so, ‘liminality opens the door to a world of contingency where events and meanings – indeed “reality” itself – can be moulded and carried in different directions’.^32^ In offering such terms as ‘permanent liminality’^33^ and ‘liminal hotspots’,^34^ recent writings in the field draw attention to the role of fluidity, flux, and epistemic interference in the constitution of everyday life as ‘once previous certainties are removed and one enters a delicate, uncertain, malleable state’.^35^

Susan Squier echoes Turner’s account of the transformation-reflection nexus in her writing on excess embryos and other objects that occupy the *limen* of human and non-human. She bridges writing on liminality with Paul Rabinow’s concept of ‘purgatory’, which she views as ‘a specific kind of liminality or in-betweenness’.^36^ For Rabinow, this purgatory is characterised by:

a chronic sense that the future is at stake; a leitmotif among scientists, intellectuals, and sectors of the public turning on redeeming past moral errors and avoiding future ones; an awareness of an urgent need to focus on a vast zone of ambiguity and shading in judging actions and actors conduct; a heightened sense of tension between this-worldly activities and (somehow) transcendent states and values; and a pressing need to define a mode of relationship to these issues.^37^

In uniting Turner’s articulation of liminality as a state of transformation with Rabinow’s diagnosis of the contemporary, Squier elicits what she views as an inherent relationship between possibility and responsibility in contemporary health research. The ontological status of novel entities often promotes a form of reflection and scrutiny in which ‘the near future and recent past’^38^ is called into play. Attention to the liminal thus helps reveal the inherent relation between knowledge, be(com)ing, and social order.

Scholarship has also explored how techno-medically mediated liminal subjectivities unsettle established moral and medico-political orders and become the catalysts for the revision of corollary modes of practice.^39^ Others from social studies of science and medicine have demonstrated an intra-relationship between *episteme* and *ontos*, which has required a rethinking of the classic human/non-human distinction. Revealing the interactive feedback loops between modes of knowing and objects of analysis in clinical and laboratory settings, this literature has focused on how physical matter, and by extension human experience, is transformed through established and nascent epistemic practices.^40^ When viewed through the lens of liminality, we might say that such betwixt and between beings threaten ‘the epistemologically neutral status of nature and its rigorous separation from society’.^41^ They do so by bringing into relief the processes of ‘purification’^42^ that separate nature from culture, normal from abnormal, living from dead. Consider, for example, the influence of the pharmaceutical industry on the delineation of new diagnostic categories of ‘abnormal populations’ in psychiatry,^43^ and the emergence of new questions as to what counts as ‘brain dead’ that accompany novel technological developments.^44^ Alongside this body of literature, we find especially useful writing on the entwined constitution of natural and socio-political orders, on how the relation between is and ought is mediated by knowledge practices,^45^ which allows us to ask: why/how are some onto-epistemologies promoted over others?

To summarise three core insights discussed above: liminality posits (1) social and ontological transformation as structuring and structured *processes and experiences* that occur in institutionally delineated time-spaces; (2) that these processes are central to the reproduction and maintenance of established organisational forms, which are otherwise threatened by liminal beings (e.g. human embryos and brain dead patients) because they reveal the constructed nature of our own being; and (3) in doing so, attention to liminality opens up new avenues for thinking about how the relationship between knowing and being in health research, and corollary experiences, are configured vis-à-vis the reproduction of extant social structures.

In contrast to the academic literature that conflates liminality with the marginal, this reading moves liminality into the centre of social and political life. It is inherent to social and political transformation – and as such, carries significant implications for regulatory design. Liminality suggests the need for a *processual*-oriented mode of regulation (not to be confused with processes *per se*) that:

- Over time, recognises the flexibility and fluidity inherent to laboratory and clinical research;
- In space, focuses on iterative interactions that adapt with new developments in science and medicine, as well as with changes in law and regulation; and
- Through experience, reflects the complete investigative endeavour and is able, for example, to guide the different involved parties through the entire research process.

We now turn to explore the potential significance of a liminal approach to health research regulation by envisioning its application to specific areas of health research, as well as its application to other kinds of actors.

## 4. Liminal ontologies

### 4.1. From the liminality of people to the liminality of things

Traditionally, liminality has been applied narrowly to people, both as individuals and communities. Though we have noted its evolution from a concept applied to ethnographic studies of ritual passages to an explanatory social theory of the modern, the species to which it applies has remained anthropological. The liminality scholar Bjorn Thomassen remarks that liminality refers to ‘how human beings experience and react to change’^46^ and that ‘experiences of liminality can be related to three different types of *subjecthood*: 1) single individuals; 2) social groups (e.g. cohorts, minorities); and 3) whole societies, entire populations, civilizations.’^47^

While Thomassen suggests that these types are broad – opening up space for possible uses beyond van Gennep’s and Turner’s understandings – in fact, this typology is rather limiting, especially when we observe how much of health research regulation focuses on objects – tissue, data, embryos, genes, and so on – rather than on the persons to whom these objects relate. Thomassen’s typology fails to account for the full tapestry of participants – both as subjects and objects – in social settings and relations, and recent developments in linking anthropology with social theory. Expanding on Squier’s contention that supernumerary embryos from artificial reproduction technologies represent ‘liminal lives’,^48^ and taking up Bruno Latour’s call to consider *all* participants at play in a social setting,^49^ we claim that liminality is not ‘merely’ a transitional, in-between state that applies to people or arguably hybrid entities like embryos or stem cells. Rather, liminality can also account for the changing relations that people have with the world and the things around them. In this sense, liminality can be applied as well to things. Things are also capable of ‘passing through’ periods or epochs of transition, leading to novel assemblages or reassemblages of understanding, behaviour, and connections to people. The paradox is that while liminality has failed hitherto to account for things, regulation all too often fails to account for experiences of subjects *in relation to* things, particularly research participants, in its drive to create regulatory objects for the purposes of the regulatory enterprise itself.

We can consider the relationship between the liminality of persons and things by observing what happens to the research protocol and attendant forms that health researchers must, under regulation, complete, and submit to multiple bodies (e.g. research ethics committees, funders, sponsors, R&D offices) when seeking approbation to commence a research project. Health research is coloured as much by researchers and other human actors as it is by the paperwork that drives the movement from the initial spark of a research question to the culmination in research performance and interpretation of results. The submitted research protocol and attendant forms are printed, stapled, folded, scanned and copied, mailed or emailed, analysed, and ruled on by authority figures who act to both adjudicate but also potentially help guide researchers through the uncertain terrain of research. At multiple instances, there can be impediments and periods of uncertainty. A form may be incomplete or inaccurate, the research design may be deemed too risky for participants, or the resources necessary to conduct the research are lacking. Can we not say that these documents or devices go through multiple transitory passages and transformations, marked by both uncertainty and the guiding (or editing) hand of a gatekeeper or steward to lead it through the passage(s) towards approbation? Liminality invokes the notion of a ‘master of ceremonies’ or ‘representative of order’ who helps guide a person through a period of transformation. In the research context, we see the importance of these regulatory actors in guiding these things *through* the liminal phase and out the other side, resolving potential crises at any moment, but also invoking their ordained power to rule on the merit of the research application’s – and not necessarily the research*er*’s – social or scientific value.

One might ask how it is possible, though, for a thing to *go through* uncertainties of the in-between. Can a thing pass ‘through’? We would respond affirmatively by drawing attention to the critical function research protocols and attendant forms – and the regulation thereof – play in health research. Latour remarks that:

if we stick to our decision to start from the controversies about actors and agencies, then *any thing* that does modify a state of affairs by making a difference is an actor – of, if it has no figuration yet, an actant.^50^

He further notes that this is not:

the empty claim that objects do things ‘instead’ of human actors: it simply says that no science of the social can even begin if the question of who and what participates in the action is not first of all thoroughly explored, even though it might mean letting elements in which, for lack of a better term, we would call *non-humans*.^51^

This claim that regulation often fails to appreciate the relationship between the liminality of persons and things, and that things can *go through* uncertainties of the in-between, is further evidenced by considering instances of contributing of one’s ‘personal data’ or ‘human tissue’ for research purposes. Both data and tissue, as we have said, are treated as ‘bounded objects’ regulated in a country like the UK by the Data Protection Act 1998 and Human Tissue Act 2004 (and Scotland’s equivalent), respectively. By ‘bounded object’ we mean things that are regulated based on whether they fall within a specific definition (in law, policy, guideline, etc., as ‘personal data’ for the Data Protection Act 1998^52^ and ‘relevant material’ for the Human Tissue Act 2004^53^), and if so, are subject to a specific corpus of rules and standards. According to both Acts, the personal data and human tissue connected to a research participant can fall *outwith* each statute’s regulatory regime *provided* that the data or tissue are anonymised (and in the case of the Human Tissue Act 2004, the research is approved by a research ethics committee). In other words, research conducted using data or tissue can be done without having to comply with numerous regulatory rules if the data or tissue are anonymised in such a way that the researcher is not in possession, and not likely to come into possession, of information that could identify the participant to whom those data or tissue otherwise appertain.

This may be satisfactory from a researcher’s perspective in foregoing ‘research red tape’ or otherwise burdensome procedures. Anonymisation has come to be a powerful tool in the researcher’s arsenal of ensuring smooth sailing through troubled regulatory waters. But from another perspective, anonymisation permitted under these regulatory regimes reflects an instance of regulatory compression in two ways. First, it fails to account for the ongoing promotion of the participant’s interests in the things that have been physically removed from them or contributed by them, yet still may have fundamental value to them (and that can change over time). For example, a participant might feel very aggrieved that ‘their’ data or tissue was used to support tobacco research even if ‘their’ data or tissue retained no linkable connection to them. By breaking (often permanently) the connection between a participant and his or her tissue or data, regulation can encourage uncertainty and anxiety – if not a ‘permanent liminality’^54^ – about the passages of these objects across sites and stages in various research projects. Anonymisation may ‘promise’ a participant privacy and security of their data and tissue (though this is a questionable promise), but it cannot promise promotion of other interests such as meaningful engagement or ongoing communication with the participant about what outcomes (research, health, or otherwise) arise from the research conducted using their data or tissue.

Second, regulatory instantiation of anonymisation reflects both an overly rigid approach to a technological process and a fallacious belief that the process is an adequate means to avoid a heightened degree of regulatory scrutiny. It is well established in the literature that anonymisation is a process performed on an object to remove its identifiability, but that as a process, it is contingent and relative, meaning it can also change over time.^55^ Indeed, anonymisation has received much scrutiny recently as a means of adequately ‘de-identifying’ genomic information. Increasingly, we have come to realise how much genomic information is re-identifiable no matter what the processes performed on them to remove traces of identifiability.^56^ However robust an anonymisation practice exists today, tomorrow it may be exposed as a broken promise of privacy.^57^ Further, there is a point at which anonymisation becomes so intense a process – a kind of race to scrub as many identifiers as possible to wipe clean all trace of connection to a person – that it renders the putatively anonymised object valueless for research.^58^ Thus, anonymising data or tissue, though regarded highly in health research regulation as a means to avoid bureaucratic scrutiny, does not absolve researchers from their legal obligations for all time coming, nor does it absolve them from ethical obligations. As anonymisation is a process and not a status, it impels researchers and regulators alike to consider how uses of these objects, even if apparently ‘disconnected’ from the participant-subject *hic et nunc*, may yet impact on the interests or sensibilities of them and their connected others (e.g. family and community members) over time. In this way we can contrast the technical *process* of anonymisation with the *processual* perspective that liminality provides. Regulation does not provide guidance about what to do here. It is true that it permits certain uses of anonymised objects, but it fails to guide us sufficiently on *how* they should be used; moreover, it fails to reflect fully the fluidity of the regulatory object – (personal)(anonymised)(personal etc.) data – over time.

In contrast, it is worth noting that the US has recently proposed in the Notice of Proposed Rulemaking (NPRM) to amend its federal Common Rule, which governs federally funded human subjects research, to expand the regulation’s definition of ‘human subject’ to include the obtaining, use, study, or analysis of biospecimens, *regardless of identifiability*.^59^ Phrased another way, biospecimens would be considered human subjects. This would bring secondary research use (i.e. use of specimens initially collected for purposes other than the currently proposed research activity) of non-identified biospecimens collected in either research or non-research settings within the purview of the Common Rule. As biospecimens would be considered human subjects, the NPRM proposes requiring informed consent for the storage and secondary research use of biospecimens, though this consent would be ‘broad’. The expressed purpose of this revision is to protect the participant’s autonomy (in other words, to let them decide whether to consent to have their biospecimens used in research) in light of risks of re-identification and possible dignitary harms, and to allow participants to have some control over future research with biological materials collected from them.

Viewed through the lens of liminality, we see this specific NPRM proposal as a positive and all-too-rare regulatory recognition of the need to recognise the fluid but bounded nature of the connection between subject with object, and to promote the participants’ interests when they contribute to the research endeavour – and which may well change over time and across various types of projects. It remains the case that secondary research use of non-identifiable (i.e. anonymised) personal data would not require consent under the Common Rule, as ‘personal data’ are not considered to be (as of yet) ‘human subjects’. This regulatory distinction between physical material and data with respect to autonomy interests and consent is questionable, yet the proposed change to include biospecimens as human subjects is a step forward. Far from reflecting semantic gymnastics (‘Biospecimens are human subjects? Impossible!’), the NPRM is instantiating a liminal reading of inseparable bond between participants and the things of theirs that they give – but not always give away – in the interests of research.

What we learn for these two examples, then, is that things – objects – in health research do not *necessarily* cause a state of affairs – such as transformations of people or events to happen (though they certainly can do so), but nor are these things inactive entities – mere passive objects – devoid of significance themselves in understanding networks of relations and assemblages. By recognising the liminality of things and their relation to participant-subjects, we come to appreciate the metaphysical shades and kaleidoscopic array of actors – *active subject-objects* – in health research (including these non-humans). We also come to appreciate how forms and other documents in health research can pass through multiple moments of transition and formation, and through their text, structure, and invocation, form a constitutive part of regulation and the processes associated with health research, be it through ethics review, requests for data access or linkage, or R&D approval from NHS bodies.

In sum, a liminality of things helps us understand how regulation and its constituent elements are part of the list of figures assembled as ‘participants’ in the research enterprise and act as a durable whole throughout processes of transition and uncertainty. Tissue and data unite with the embodied participant as One. A liminality of things thus enables us to recognise the connections between regulatory objects and the human subjects from whom they are derived, and between regulatory devices (such as anonymisation and consent) and the human subjects that modify their state of affairs. Less positively, it can demonstrate how compressed regulation centres on ‘bounded objects’ and disregards the transformations and transforming expectations and interests of these active subject-objects. We come to recognise, then, how many more actors and subjectivities are in play in the course of any given health research project, irrespective of whether regulation recognises this. And indeed, it often does not, as the processual case of the human embryo makes even more clear.

### 4.2. A processual case: the human embryo

The UK’s regulatory approach to human embryos is one way in which we might highlight that the law regulates on the basis of several underlying scientific and ethical assumptions. Normatively, the moral status of the human embryo is viewed in the singular, especially by law. This is not an adequate reflection of biological reality. Gaps exist between the regulatory categories that the law has created, such as between gamete and embryo; embryo and foetus; and foetus and baby. Moreover, even though law attempts to clarify these categories of being, the lines remain blurred as demonstrated by ongoing debates, for example, concerning embryonic stem cell research or time-limits on abortion. Yet biological growth of the embryo, from conception, is manifestly a continuous process not a set of several ‘levels’ of being, as the law attempts to prescribe. To what extent, then, is it the law’s place to ‘handle’ or ‘remedy’ the ethical questions that surround the human embryo, when the underlying assumptions upon which the legal edifice is built are not concrete?

The concept of liminality once again provides useful insight here. Let us consider the link between liminality, the embryo, and morality. The moral status that we afford the embryo informs the law, and the ways in which we represent biology in law have an important effect on the role we, as a society, afford to biology. Arguably, morality and law currently depict ‘the embryo’ as a unitary entity. Is there any other manner in which we could view ‘the embryo’, and if there is, what are the consequences of doing so?

Regulation of the embryo in the UK, under the Human Fertilisation and Embryology Act (HFEA) 1990 (as amended), embodies the ‘compromise position’, as recommended by the Warnock Committee. This position has been described as affording ‘respect’ for the human embryo.^60^ While this position does not give the embryo a ‘lower’ moral status equivalent to that which we afford to human tissue or other products of the human body, it is also not afforded personhood:

the compromise position is not concerned to protect individual human embryos, since these will ultimately be destroyed, but is instead directing towards protecting the symbolic value of early human life.^61^

In this way, the compromise position seems to recognise the embryo’s transitional state, from cell(s), to human being (or ‘person’).^62^ Yet, although the compromise position embraces the embryo’s liminal state to some extent, it does not do so fully. Despite governing two potentially distinct processual pathways on which the embryo might find itself – namely, reproduction or research – the HFE Act 1990 (as amended) covers these two contexts within the same piece of regulation.

Embryo regulation is a form of static, hard law, and the question of the status of the embryo – as laid down by Warnock – has not been revisited since the creation of the original HFE Act in 1990. Even the debacle over cloned embryos was effectively shut down by the House of Lords in 2005 in *Quintavalle*.^63^ This is arguably an example where potential for new, more public input into the regulation of embryos has been blocked, thus maintaining the rigidity and inflexibility of one of the core focuses of the HFE Act, viz., the status of the embryo. Accordingly, there is little in the way of substantive feedback loops,^64^ and the statute itself is quite inflexible in this regard. Although there are, quite commendably, regular consultations on certain proposed incremental amendments to the Act (for example on mitochondrial replacement therapy in 2014 leading to 2015 Regulations),^65^ there is little other outlook for this fundamental issue to be addressed, and it is certainly not done so in these consultations. The issue at hand here is, therefore, that the status of the embryo has been made entirely inflexible by the law.

There are at least two reasons why regulating the embryo through static, hard law is problematic. First, scientific understandings, and ethical attitudes towards them, are ever-evolving. Second, as aforementioned, the evolution from gamete, to embryo, to foetus, to child is itself processual (and not a set of entirely separable ‘stages’). Thus, embryos are regulated in an inflexible manner not only in terms of the lack of statutory re-visitation, but also in terms of the static, and stage-like manner in which the law tends to segment this biological reality. Once again, regulation creates a series of ‘bounded objects’ to be the subject of its attention.

For humanities scholar Susan Merrill Squier, mentioned above, current embryonic stem cell research has created ‘a liminal space in which the meaning and value of the human embryo is in flux’.^66^ Science has enabled us to see biological processes more clearly than ever. Nonetheless, this increased knowledge and scrutiny has come paired with an increased scientific awareness of the different ‘uses’ that human embryos may have:

Human embryos are no longer defined only as those things that become human; now, human embryos have the potential to become other things, such as research subjects, stem cell repositories, and facilitators of therapeutic cloning research. Their value is increased by their enhanced utility. For Squier, biotechnical innovations and their accompanying discursive constructions alter the liminal space in which science and human bodies interact. The liminal space can be seen as a field of knowledge production where the contestation of claims happens in numerous ways … .^67^

Thus, the transitional state that the embryo occupies is not invariant; indeed it may be subject to (at least) two outcomes: reproductive purposes, and research purposes. Interestingly, Devaney refers to the ‘dual reproductive identity’ of the embryo:

Thus, at the point embryo progenitors make their decision, their intention helps to shape the embryo’s legal status, indicating the context within which its value (whether reproductive, therapeutic or other) should be determined. This has been termed the ‘dual reproductive identity’ of embryos consisting of past attempts to conceive a child and the future capacity of science to transform the vital power of individual cells into colonies of regenerative cells.^68^

The embryo itself is a liminal entity, aside from the liminal states that the law has created for it by delineating categories of being. It is an entity in an ongoing process of ‘becoming’. Moreover, the transitory state of the embryo has been recognised in other cultures, for example in *mizuko kuyō*, a Japanese tradition for grieving miscarriages that recognises liminality in embryonic life.^69^ By virtue of this process of becoming, it makes little sense to categorise it as a singular entity. Moreover, not only does the ontology of the embryo change as it moves into different time-spaces, but it is also important to recognise that the ability to engage with the therapeutic possibilities of the embryo for research is dependent on seeing it as a fluid entity that is co-produced at the intersection of regulation, biology, and laboratory approaches technologies. The embryo as a resource for hESC research, for example, ‘is defined by its being “not yet” something else, that is a stem cell is defined more in terms of a biological possibility – its pluripotency – than a well-defined actuality’.^70^

The obvious next question, however, is So What? For one thing, the failure of law to reflect these realities is yet another instance of regulatory compression and potentially sub-optimal operation of the regulatory infrastructure itself. Beyond this, there is also, arguably, a third state, described below, that embryos occupy at a certain point in time, perhaps even more liminal than the latter two. This is the embryo with an unknown future. Thus, at least three liminal states of the embryo may be said to exist:

1. Where the embryo is to be used for reproductive purposes.
2. Where it is going to be used in research.
3. Where it has an uncertain future, e.g. when being used for PGD, its future could be either state 1 or 2. This reflects Turner’s description of a state of ‘pure possibility’.^71^

Liminality thus makes evident the ontological questions related to the moral status of the embryo, and, following on from this, issues with regulating the embryo as a singular object. Moreover, liminality is not only relevant here in terms of the embryo’s ‘in-between’ state, but also by virtue of it being an entity in a process of *becoming*. This has implications on multiple levels, for example embryos, donors, and parents, to name a few. Pertinent to this context, the liminal quality of embryos has the potential to greatly influence scientific researchers. A study by Svendsen and Koch challenges the idea that ‘spare’ embryos are a biological fact, and rather are constituted by the decision-making of researchers.^72^ This ‘ongoing fact-making’ reveals a network of relationships and conflicts in which researchers are involved and through which the embryo passes and is variously assembled and disassembled.

What this entity *is* becoming, is, as aforementioned, variable. This outcome is of course largely at the whim of external actors in this context (with the notable exception that in the context of ‘natural reproduction’ the different outcomes of the embryo may also be affected by biological factors not in control of the mother, or others).^73^ The notion of subject and object is once again important here. Where the embryo is given a ‘human’ path (as a consequence of occupying state 1), its treatment as a subject, rather than as an object, could be said to intensify.^74^ Conversely, where the embryo occupies state 2, they are arguably ‘dehumanised’. The treatment of the embryo as a subject *or* object under law (or perhaps on a scale from subject to object) becomes clearer when embryos are separated into the three liminal states listed above. However, as with our earlier subject-object discussion relating to ‘personal data’ and ‘human tissue’, our analysis on the liminal statuses of the embryo – on any path – suggests that connections with subjects already enjoying full personhood, namely the biological creators of the embryo, ought not to be too easily or quickly severed.

The three states identified herein are problematic for *the* status of ‘the embryo’ under law. This analysis suggests that there are good reasons to explore more fully whether it is sustainable to continue to regulate as if there was a unitary entity. Liminality has great disruptive potential in this area in its capability to highlight the static (and false) scientific and ethical assumptions that the law embodies when regulating the human embryo. Liminality forces us to recognise the differences in status that the embryo may experience depending on the paths upon which it is put. With changeable contexts come fluid boundaries. In particular, liminal boundaries are fluid because contexts can change. As stated above, an embryo used in PGD might be used for research *or* be used for reproduction. The definitions that we use for differing stages of the embryonic process, and the different contexts that apply to those, are themselves open to change. Furthermore, ‘embryo’ is a temporal-based definition; it describes an entity’s place in a moment and within a process. Thus the fluidity shown here exemplifies yet another kind of liminality: it is a liminality itself defined by the embryo being suspended in a threshold or particular space in time: a space of uncertainty.

The liminal states and the subject/object dyad are important for the future of artificial reproduction and embryo research because the notion of ‘the moral status of the embryo’ underpins the entire legal architecture of human reproductive regulation. A liminal perspective suggests, however, that at best, the law may be perpetuating a moral myth, and at worst, the compressed regulatory regime is fundamentally flawed.

## 5. Thresholds and spaces in time

### 5.1. Liminality as a spatial metaphor

The case study of the embryo demonstrates the ways in which liminality can be used to analyse processual elements in the definition of biological entities. However, as Van Gennep’s initial formulation describes processes of change in space and time,^75^ another way of thinking about liminality is to focus on the spatial and temporal aspects of the processes under consideration. This section will discuss ways of using spatial approaches in our thinking about health research regulation.

One way of achieving this is to consider how liminality might be combined with approaches from Science and Technology Studies (STS) that use geographic and cartographic metaphors. Canonical examples of such approaches include ‘boundary objects’,^76^ ‘boundary work’,^77^ ‘situated knowledge’,^78^ ‘standpoints’,^79^ and ‘trading zones’.^80^ In the rest of this section, we will consider two such conceptual metaphors, boundary objects^81^ and boundary work, as they have been very influential, inter alia, due to their use to understand processes of regulation. Both of these approaches rely on ideas of interpretative flexibility, i.e. that objects and ideas can have multiple uses and/or meanings depending on the social context in which they are used.^82^ We will argue that, in combination with ideas about liminality, these spatial approaches provide clearer understandings of regulatory regimes by drawing attention to empty spaces, gaps, and overlaps in regulatory processes.

The idea of boundary objects was first developed by Susan Leigh Star and James Griesemer in their historical study of the groups working at the Berkeley Museum of Vertebrate Biology, where amateur collectors, professional scientists, philanthropists and administrators managed to collaborate productively in the absence of consensus.^83^ Leigh Star and Griesemer analysed the role of objects, and, in particular, standardised specimen records:

Records of the specimens had different meanings for the different groups of actors, but each group could contribute to and use these records. The practices of each of the groups could continue intact, but the groups interacted via record-keeping.^84^

To function successfully as a boundary object and act as a bridge between social groups, an entity has to be sufficiently flexible to serve the interests of each group yet robust enough to maintain its identity in different social contexts. The concept of boundary object has been widely used within STS, but also criticised for a lack of precision (can anything be a boundary object?). However, in the regulatory context it directs analytic attention towards the sharing of procedures and entities, rather than understandings and values. Consideration of the specific groups that use particular boundary objects, and the different ways in which they understand these objects can also be one way of drawing attention to the marginalisation of publics’ participation in health research and disregard of their concerns. For example, since they are relatively stable in form and used by different groups within research, consent forms can be understood as boundary objects. By asking about their use, a study of consent forms could be used to investigate what different groups understand by participation in particular biomedical research projects, and whether processes of obtaining consent addresses their concerns about the research and its outputs.

As Leigh Star points out, interpretive flexibility is not unique to boundary objects, and their organisational structure and scale also need to be considered: if boundary objects are used very consistently at a larger scale, they turn into new entities such as infrastructures or standards.^85^ There has been an increasing interest in the creation and maintenance of standards by means of entities such as patient records,^86^ definitions of obesity and overweight using body mass index categories,^87^ and international classifications of disease.^88^ A liminal approach complements this scholarship because it highlights the effects of rigid or static classificatory systems in the fluid contexts of biomedical research and regulation. Consider again the example of hESC research, where ‘standardization [was used] to stabilize human embryonic stem cells (hESCs) as biological objects and in building the stem cell field itself’.^89^

The second concept is boundary work. In outlining constructivist approaches to the study of science, historian of science, Thomas Gieryn, developed an influential metaphor to describe how:

[t]he boundaries of science are episodically established sustained, enlarged, policed, breached, and sometimes erased in the defense, pursuit, or denial of claims of epistemic authority. As knowledge makers seek to present their claims or practices as legitimate (credible, trustworthy, reliable) by locating them within ‘science,’ they discursively construct for it an ever changing arrangement of boundaries and territories and landmarks, always contingent on immediate circumstances.^90^

Gieryn labels the activities involved in building, maintaining, adapting and policing these discursive perimeters ‘boundary-work’ which takes place ‘as people contend for, legitimate, or challenge the cognitive authority of science’.^91^ Such tasks are fundamental to the credibility of scientific knowledge. Debates about the credibility of a particular scientist or areas of scientific research are usually based on norms of scientific explanation (does a phenomenon contradict current models and theories?), scientific practice (is a process replicable?) or behaviour (is a researcher acting from financial or political motives?). Flexible interpretation of such norms provides endless rhetorical possibilities, depending on specific context and purpose. A contemporary example of the shifting boundaries between science and non-science is provided by the definition of complementary and alternative medicine: homoeopathy and herbalism are interesting contrasting examples here. The underlying mechanisms of homeopathy are now dismissed as scientifically implausible, whereas pharmaceutical research investigates the physiological effects of phytochemicals and uses them as the basis of new drugs such as artemesinin compounds to treat malaria.

The concept of boundary work has been used to understand the use of research evidence in regulatory processes. Sheila Jasanoff argues that advisory (or policy-orientated or regulatory) science is ‘a hybrid activity that combines elements of scientific evidence and reasoning with large doses of social and political judgement.’^92^ Boundary work is central to this understanding of the interaction between scientific expertise and policymaking. In her case studies, Jasanoff demonstrates how, in order to exempt ‘technical’ matters from political control, expert scientists define the boundary between science and policy:

By drawing seemingly sharp boundaries between science and policy, scientists in effect post ‘keep out’ signs to prevent non-scientists from challenging or reinterpreting claims labelled as ‘science’. The creation of such boundaries seems crucial to the political acceptability of advice. When the boundary holds, both regulators and the public accepts the experts’ designation as controlling, and the recommendations of advisory committees, whatever their content, are invested with unshakeable authority.^93^

However, this fluid boundary can also be used by expert scientists to define the limits of their authority and avoid their recommendations becoming politically controversial. For example, scientific advice about the relationship between diet and health has often been highly charged due to the operation of extensive commercial interests in this area and the potentially wide-ranging political implications of public health policy. Researchers serving on expert committees in this field use wider or narrower definitions of obesity science (and therefore their expertise) depending, for example, on whether they are trying to explain rising rates of excess body weights, or offer politically palatable solutions to the problem.^94^ The pervasiveness of boundary work suggests that fluidity is an inherent aspect of recourse to scientific expertise – who counts as an expert and whether the issue is primarily one of science or, for example, ethics are often contested points. It also suggests that in certain contexts regulatory actors will make use of fluidity if it is available to them.

As outlined above, many uses of the concept of liminality focus on the processual, transformative experiences that take place in-between boundaries and thresholds. Here, liminality is often understood as occurring at the edges or margins of particular places and times.^95^ However, when analysing regulatory processes, a focus on margins is not very helpful. In this section, we have presented an alternative, and complementary approach that understands liminality as centrally concerned with movement across boundaries and thresholds. Mapping these changing boundaries – between science and non-science – and the objects – such as patient record or consent forms – that move across them, can therefore be used to highlight gaps in regulatory regimes. These include the unregulated spaces created by experimental treatments and new medical technologies, the empty spaces where regulatory regimes have historically been weak, and overlaps where entities, such as reproductive tissues, are subject to more than one form of regulation. Bringing cartographic approaches from STS (and other disciplines) into dialogue with liminality thus allows for a comprehensive and illuminating spatially-informed analysis of particular regulatory regimes.

### 5.2. Temporality and being(s)

In this section we return to questions of temporality, using two examples to illustrate how regulatory compression engenders research in ways that might hinder research and fail to attend to the interests of research participants and/or marginalise publics’ participation both in research and in its regulation. These two examples are the governance of emerging technologies and the concept of social value as an ethical requirement for health research. Both examples illustrate how the temporal rigidity of existing regulatory mechanisms constrain the values and voices that are critical to successful governance.

#### 5.2.1. Precautionary principle

Emerging technologies require the creation of ‘possible products’ and ‘possible futures’ for their funding and their regulation. These may or may not materialise. Regulation and governance, however, depend on the creation of these potential products and the potential (positive and negative) futures associated with them. Much governance in this context is not, however, about regulating entities but rather their potential, constantly creating and re-creating the spaces between and across disciplines, as well as the purview and limits of associated regulation. As new, empty spaces are created, we are constantly faced with an urge to contain and control the unknown (since that is what regulation often implies). This can be seen, for example, in current discussions surrounding gene editing.^96^ Questions in this area range from, ‘Are any new social, ethical, or legal concerns raised by gene editing?’, and ‘If so, is a precautionary form of regulation required?’, to ‘What is the object of regulation – a risk, a product?’ Alternatively, is this merely another emerging technology that can, or should be, compressed to ‘fit’ within existing regulatory frameworks? We suggest that this is another instance of a liminal space being created cotemporaneously with the uncertainties generated by new and emerging technologies. Liminality suggests that there is an urgent need for someone (or something – perhaps some form of regulation) to lead us through.

Risk-based evaluation is often a predominant feature of such empty liminal spaces. Interestingly, however, such spaces raise important ethical questions as they become sites for battles of power, for example, between environmentalists and the industries they oppose,^97^ as well as battles of values. The precautionary principle, or more generally the idea of precaution, is one such example. It assumes two things: that the status quo is good and the future is risky; and that caution is a virtue and the potential problems lie with changing the status quo.^98^ On the one hand, such spaces can become the site for a democratic process (or at least for an important involvement of publics), but on the other hand, power can also quickly shift into techno-scientific and regulatory hands by trying to contain the values created in these spaces by ‘science’-based value judgments, e.g. by containing fear, hope, power and accountability within the seemingly value-neutral territory of risk and probability and into the hands of ‘experts’. An appeal to the precautionary principle might initially appear to include the anxieties of concerned publics, but in fact its regulatory expression (its dependence on risk-expertise and calculations) might serve to further alienate publics, leading rather to regulatory compression, as we have suggested above. Calls to use the precautionary principle for new technologies,^99^ and lately, a call for a moratorium for gene editing,^100^ have an apparent purpose of addressing the values of publics, without necessarily ceding the power from the hands of ‘experts’. As a result, these liminal spaces are kept strictly within the remit of science and regulation, allowing tools such as the precautionary principle to become political tools rather than tools of democratically-informed governance. The rest becomes a matter of public perception and public engagement. It need not be thus. Liminality invokes a realm of possibility, but equally because of inherent uncertainties, the liminal space can be open to misuse and abuse.

#### 5.2.2. Social value

A second and final example of the tension between the temporal limitations stemming from existing regulatory mechanisms – and the ideal ethical values that we wish to see represented in science and scientific governance – is illustrated by the contemporary bioethical debate surrounding ‘social value’ in research.^101^ Although not a new concept in bioethics, the concept of social value seems to have gained some traction lately as part of the ethical evaluation of research (especially clinical research) and of specific research protocols. On the one hand, social value, referred to as ‘the prospect of generating the knowledge and/or the means necessary to protect and promote people’s health’^102^ is meant to act as a primary ethical justification for research.^103^ Under this reading, social value acts as a ‘threshold device’, a mechanism of transition for the research proposal, purporting to transform the research question or design into something worthwhile, with other ethical considerations following. If research does lead to something of value, the question remains at which point the results of research become valuable.^104^ While it may be possible to establish some objective accounts as to what counts as valuable, and value will constantly be created and redefined as research progresses, the required feedback loops are often not present in current governance structures, which as noted above are often ‘fixed and consequently binding’. This, once again, is a liminal and thus processual enterprise.

The process of ethics review is an example of rigidity within the governance structure, in which the concept of social value remains trapped within an institutionally delineated time-space both temporally (as part of ethics approval prior to the commencement of a clinical study, for example), and also discursively (within the discussion of risk assessment). This might be seen as a form of permanent liminality – where uncertainties and anti-structures prevail. More importantly, as an analytical tool, liminality leads us to question the very role of social value in all of this. Arguably, the demands of social value ask us to never leave this feedback loop; to constantly revisit, question and appraise both what might be considered valuable or not, and to revisit our standards of what is seen as valuable. Once we realise this, we might look for alternative approaches. For example, the creation of more flexible and reflexive governance practices, with feedback loops and iterative forms of collaborative knowledge production regulation, may thus allow us to unleash the potential of ‘social value’ as a concept.

## 6. Conclusion

We began this article by outlining current approaches to health research regulation. We offered the term ‘regulatory compression’ to characterise the effects of these approaches on research practices. With its focus on transformation and the ‘in-between’, liminality allows us to see how extant regulatory frameworks rely on a silo-based approach to classifying research objects that: (1) limits the flexibility necessary in clinical and laboratory research; (2) results in the emergence of unregulated spaces that lie between the bounded regulatory spheres; and (3) curtails modes of public participation in the health research enterprise. As a concept, liminality sheds new light on the boundary-crossing inherent to health research and thus calls for a radical reimagining of regulatory approaches. Going forward, we suggest there is a need to develop the notion of *processual regulation*. This is far more than a mere focus on processes in regulation. Rather, as outlined above, such an approach requires a temporal-spatial examination of regulatory spaces and practices as these are experienced by all actors, including the relationship of actors with the objects of regulation. In particular, we suggest that processual-oriented regulation has the following features:

- Over time, it recognises the flexibility and fluidity inherent in laboratory and clinical research;
- In space, it focuses on iterative interactions that adapt with new developments in science and medicine, as well as with changes in law and regulation; and
- Through experience, it reflects the complete investigative endeavour and is able, for example, to guide the different involved parties through the entire research process.

There are a number of questions that will need to be answered when designing a processual-oriented mode of regulation. Questions that we can identify at present include:

- How can a regulatory system that recognises flexibility also demonstrate its trustworthiness to researchers and research participants alike?
- How does processual regulation dovetail with the common commitment to deliver proportionate regulation; that is, would such an approach increase or decrease the bureaucratic labour involved in health research?
- Who gets to decide what constitutes ‘the entire research process’, and how?

The premise of this article is that much is lost in current regulatory approaches for the failure to recognise and acknowledge a reality of human experience: that liminality is all around us and experienced on a daily basis. We have demonstrated the applicability of liminality to the health research enterprise in a number of contexts and we have developed a sophisticated conceptual toolkit in the process. Our core contribution has been to suggest that the liminal regulatory spaces that already exist are under-theorised and are operating sub-optimally for the want of the insights that liminality can bring. The reality is that liminal spaces exist everywhere. We encourage the reader to draw on our analysis to recognise these in myriad contexts and to deploy corollary perspectives in navigating all such spaces that emerge ‘in-between’.

## Footnotes

1 Frank Vibert, *The New Regulatory Space: Reframing Democratic Governance* (Edward Elgar, 2014).

2 Leigh Hancher and Michael Moran, *Capitalism, Culture and Economic Regulation* (Oxford University Press, 1989).

3 Bronwen Morgan and Karen Yeung, *An Introduction to Law and Regulation: Text and Materials* (Cambridge University Press, 2007).

4 Surendra Arjoon, ‘Striking a Balance Between Rules and Principles-based Approaches for Effective Governance: A Risk-based Approach’ (2006) 68 *Journal of Business Ethics* 53.

5 Nayha Sethi, ‘Reimagining Regulatory Approaches: On the Essential Role of Principles in Health Research Regulation’ (2015) 12 *SCRIPTed* 91.

6 Julia Black, ‘The Emergence of Risk-based Regulation and the New Public Risk Management in the United Kingdom’ [2005] *Public Law* 512.

7 Robert Baldwin, ‘Regulation: After Command and Control’ in Keith Hawkins (ed), *The Human Face of Law* (Oxford University Press, 1997) 65–84.

8 Sheila Jasanoff, *States of Knowledge: The Coproduction of Science and the Social Order* (Routledge, 2004); Jennifer Reardon, *Race to the Finish: Identity and Governance in the Age of Genomics* (Princeton University Press, 2004).

9 Contrast, Roger Brownsword, ‘In the Year 2061: From Law to Technological Management’ (2015) 7 *Law, Innovation and Technology* 1.

10 See World Medical Association, *Declaration of Helsinki* (World Medical Association, 2013); Council for International Organizations of Medical Sciences (CIOMS), *International Ethical Guidelines for Biomedical Research Involving Human Subjects* (CIOMS, 2002). See also Richard Ashcroft, ‘The Declaration in Helsinki’ in Ezekiel Emanuel and others (eds), *The Oxford Textbook of Clinical Research Ethics* (Oxford University Press, 2008) 141–48.

11 Arthur Kleinman, *Writing at the Margin: Discourse Between Anthropology and Medicine* (University of California Press, 1995); Paul Brodwin, ‘The Coproduction of Moral Discourse in US Community Psychiatry’ (2008) 22 *Medical Anthropology Quarterly* 127.

12 Charles Rosenberg, ‘Meanings, Policies, and Medicine: On the Bioethical Enterprise and History’ (1999) 128 *Daedalus* 27; Carl Elliot, ‘Fear and Loathing in Bioethics’ (2016) 6 *Narrative Inquiry in Bioethics* 43.

13 Brodwin (n 11).

14 Samuel Taylor-Alexander, ‘Adjacent Temporalities: Making Medico-Scientific Futures and the Present’ (2015) 1 *Engaging Science, Technology, and Society* 67.

15 J Benjamin Hurlbut, ‘Remembering the Future: Science, Law, and the Legacy of Asilomar’ in Sheila Jasanoff (ed), *Dreamscapes of Modernity: Sociotechnical Imaginaries and the Fabrication of Power* (University of Chicago Press, 2015) 126–51.

16 Finnuala Kelleher, ‘The Pharmaceutical Industry’s Responsibility for Protecting Human Subjects of Clinical Trials in Developing Nations’ (2004) 38 *Columbia Journal of Law and Social Problems* 67.

17 Linsey McGoey, ‘The Logic of Strategic Ignorance’ (2012) 63 *The British Journal of Sociology* 533.

18 Ibid, 553.

19 Sheila Jasanoff, *Science and Public Reason* (Routledge, 2012); Krishanu Saha and J Benjamin Hurlbut, ‘Treat Donors as Partners in Biobank Research’ (2011) 478 *Nature* 312.

20 Paul Rabinow and Gaymon Bennett, *Designing Human Practices: An Experiment With Synthetic Biology* (University of Chicago Press, 2012); Robin Williams, ‘Compressed Foresight and Narrative Bias: Pitfalls In Assessing High Technology Futures’ (2006) 15 *Science as Culture* 327.

21 Rosenberg (n 12); Elliot (n 12).

22 Graeme Laurie and Shawn Harmon, ‘Through the Thicket and Across the Divide: Successfully Navigating the Regulatory Landscape in Life Sciences Research’ in Emilie Cloatre and Martyn Pickersgill (eds), *Knowledge, Technology and Law* (Routledge, 2014) 121–36.

23 Rabinow and Bennett (n 20).

24 Jane Kaye and others, ‘ELSI 2.0 for Genomics and Society’ (2012) 336 *Science* 673.

25 Saha and Hurlbut (n 19).

26 Barbara Prainsack and Alena Buyx, *Solidarity: Reflections on an Emerging Concept in Bioethics* (Nuffield Council on Bioethics, 2011).

27 Arnold van Gennep, *The Rites of Passage* (University of Chicago Press, 2011[1909]).

28 Victor Turner, *Ritual Process: Structure and Anti-Structure* (Cornell University Press, 1969).

29 Ibid, 95.

30 Ibid, 240.

31 Ibid, 7.

32 Bjorn Thomassen, *Liminality and the Modern: Living Through the In-Between* (Ashgate, 2014) 7.

33 Árpád Szakolczai, ‘Liminality and Experience: Structuring Transitory Situations and Transformative Events’ (2009) 2 *International Political Anthropology* 141.

34 Paul Stenner, ‘Liminality: Un-Wohl-Gefühle und der affective turn’ in Elisabeth Mixa and others (eds), *Un-Wohl-Gefühle: Eine Kulturanalyse gegenwärtiger Befindlichkeiten* (transcript Verlag, 2016) 45–68.

35 Szakolczai (n 33) 172.

36 Susan Merrill Squier, *Liminal Lives: Imagining the Human at the Frontiers of Biomedicine* (Duke University Press, 2004) 8.

37 Paul Rabinow, *French DNA: Trouble in Purgatory* (University of Chicago Press, 1999) 17–18.

38 Paul Rabinow, *Anthropos Today* (Princeton University Press, 2003) 55.

39 Robert Barrett, ‘The “Schizophrenic” and the Liminal Persona in Modern Society’ (1998) 22 *Culture, Medicine and Psychiatry* 465; Jean Jackson, ‘Stigma, Liminality, and Chronic Pain: Mind-Body Borderlands’ (2005) 32 *American Ethnologist* 332; Squier (n 36).

40 Karen Barad, *Meeting the Universe Halfway: Quantum Physics and the Entanglement of Matter and Meaning* (Duke University Press, 2007); Annemarie Mol, *The Body Multiple: Ontology in Medical Practice* (Duke University Press, 2002); Charis Thompson, *Making Parents: The Ontological Choreography of Reproductive Technologies* (MIT Press, 2005); Steve Woolgar and Javier Lezaun, ‘The Wrong Bin Bag: A Turn to Ontology in Science and Technology Studies?’ (2013) 43 *Social Studies of Science* 321.

41 Margaret Lock, *Twice Dead: Organ Transplants and the Reinvention of Death* (University of California Press, 2002) 41.

42 Bruno Latour, *We Have Never Been Modern* (Harvard University Press, 1993).

43 Allan Young, *The Harmony of Illusions: Inventing Post-Traumatic Stress Disorder* (Princeton University Press, 1997); Andrew Lakoff, *Pharmaceutical Reason: Knowledge and Value in Global Psychiatry* (Cambridge University Press, 2005).

44 Lock (n 41); Squier (n 36).

45 Jasanoff (n 8); Reardon (n 8).

46 Thomassen (n 32) 1.

47 Ibid, 89.

48 Squier (n 36).

49 Bruno Latour, *Reassembling the Social: An Introduction to Actor­Network­Theory* (Oxford University Press, 2005).

50 Ibid, 71.

51 Ibid, 72.

52 Data Protection Act 1998, s 1(1).

53 Human Tissue Act 2004, s 53.

54 Szakolczai (n 33).

55 Deryck Beyleveld and David Townend, ‘When is Personal Data Rendered Anonymous? Interpreting Recital 26 of Directive 95/46/EC’ (2004) 6 *Medical Law International* 73; Nayha Sethi and Graeme Laurie, ‘Delivering Proportionate Governance in the Era of eHealth: Making Linkage and Privacy Work Together’ (2013) 13 *Medical Law International* 168.

56 Yaniv Erlich and Arvind Narayanan, ‘Routes for Breaching and Protecting Genetic Privacy’ (2014) 15 *Nature Reviews Genetics* 409; Melissa Gymrek and others, ‘Identifying Personal Genomes by Surname Inference’ (2013) 339 *Science* 321.

57 Paul Ohm, ‘Broken Promises of Privacy: Responding to the Surprising Failure of Anonymization’ (2010) 57 *UCLA Law Review* 1701.

58 Council of Canadian Academies, *Accessing Health and Health-Related Data in Canada* (Council of Canadian Academies, 2015).

59 United States Department of Health, ‘NPRM for Revisions to the Common Rule’ (8 September 2015) www.hhs.gov/ohrp/humansubjects/regulations/nprmhome.html (accessed 26 January 2016).

60 Although later, Baroness Warnock pointed out that one cannot ‘respect’ something you ‘throw away’, so she instead suggested that it embodies the principle of ‘non-frivolity’.

61 Emily Jackson, *Medical Law: Text, Cases, and Materials* (Oxford University Press, 2013) 638.

62 Squier (n 36).

63 
*R v S of S for Health ex parte Quintavalle* (on behalf of Pro-Life Alliance) [2003] 2 WLR 692 House of Lords.

64 As aforementioned, ‘feedback loops’ are outputs of a regulatory system routed back as inputs to the various actors implicated in the enterprise.

65 See e.g. Human Fertilisation and Embryology Authority, ‘Mitochondria Replacement Consultation: Advice To Government’ (2013) www.hfea.gov.uk/docs/Mitochondria_replacement_consultation_-_advice_for_Government.pdf; Nuffield Bioethics, ‘Draft Regulations On Mitochondrial Replacement Techniques Echo Council Recommendations: Nuffield Bioethics’ (2014) www.nuffieldbioethics.org/news/draft-regulations-mitochondrial-replacement-techniques-echo-council-recommendations; Nuffield Council on Bioethics, ‘Novel Techniques For The Prevention Of Mitochondrial DNA Disorders: An Ethical Review’ (2012) www.nuffieldbioethics.org/wpcontent/uploads/2014/06/Novel_techniques_for_the_prevention_of_mitochondrial_DNA_disorders_compressed.pdf?; Department of Health, ‘Mitochondrial Donation: A Consultation On Draft Regulations To Permit The Use Of New Treatment Techniques To Prevent The Transmission Of A Serious Mitochondrial Disease From Mother To Child’ (2014) www.gov.uk/government/uploads/system/uploads/attachment_data/file/285251/mitochondrial_donation_consultation_document_24_02_14_Accessible_V0.4.pdf; Department of Health, ‘Mitochondrial Donation: Government Response To The Consultation On Draft Regulations To Permit The Use Of New Treatment Techniques To Prevent The Transmission Of A Serious Mitochondrial Disease From Mother To Child’ (2014) www.gov.uk/government/uploads/system/uploads/attachment_data/file/332881/Consultation_response.pdf (all accessed 26 January 2016).

66 Mary Ingram-Waters, *Unnatural Babies: Cultural Conceptions of Deviant Procreations* (University of California, PhD thesis, 2008) 75.

67 Ibid.

68 Sarah Devaney, *Stem Cell Research and the Collaborative Regulation of Innovation* (Routledge, 2013) 15–16.

69 Elizabeth Harrison, ‘Women’s Responses to Child Loss in Japan: The Case of *mizuko kuyō*’ (1995) 11 *Journal of Feminist Studies in Religion* 67.

70 Andrew Webster and Lena Eriksson, ‘Governance-by-Standards in the Field of Stem Cells: Managing Uncertainty in the World of “Basic Innovation”’ (2008) 27 *New Genetics and Society* 99, 105.

71 Turner (n 28).

72 Mette Svendsen and Lene Koch, ‘Unpacking the “Spare Embryo”: Facilitating Stem Cell Research in a Moral Landscape’ (2008) 38 *Social Studies of Science* 93.

73 In highlighting the impact of hESC materiality on research practice, for example, Eriksson and Webster write: ‘In public domains, hESCs tended to be described as comparatively stable and compliant entities  …  Technicians would testify that the private stem cell was of a different disposition entirely – difficult to maintain it in an undifferentiated state, let alone to push it down specific developmental trajectories’: Lena Eriksson and Andrew Webster, ‘Standardizing Work as a Recursive Process: Shaping the Embryonic Stem Cell Field’ (2015) 34 *New Genetics and Society* 72 at 76.

74 For example, under the Abortion Act.

75 Van Gennep (n 27).

76 Susan Leigh Star and James Griesemer, ‘Institutional Ecology, “Translations”, and Boundary Objects: Amateurs and Professionals in Berkeley’s Museum of Vertebrate Zoology’ (1989) 19 *Social Studies of Science* 387.

77 Thomas Gieryn, *Cultural Boundaries of Science* (University of Chicago Press, 1999).

78 Donna Haraway, *Modest_Witness@Second_Millennium.FemaleMan_Meets_OncoMouse: Feminism and Technoscience* (Routledge, 1997).

79 Sandra Harding, *The Science Question in Feminism* (The Open University Press, 1986).

80 Peter Galison, ‘Trading Zone: Co­ordinating Action and Belief’ in Mario Biagioli (ed), *The Science Studies Reader* (Routledge, 1999) 137–60.

81 Not to be confused with ‘bounded objects’ as discussed in previous sections referring to legal categories created for regulatory purposes such as ‘human tissue’ and ‘personal data’.

82 Sergio Sismondo, *An Introduction to Science and Technology Studies* (Blackwell, 2004); Susan Leigh Star, ‘This is Not a Boundary Object: Reflections on the Origin of a Concept’ (2010) 35 *Science, Technology & Human Values* 601.

83 Leigh Star and Griesemer (n 76).

84 Sismondo (n 82) 148.

85 Leigh Star (n 82) 602.

86 Stefan Timmermans and Marc Berg, *The Gold Standard: The Challenge of Evidence-based Medicine and Standardization in Health Care* (Temple University Press, 2003).

87 Isabel Fletcher, ‘Defining an Epidemic: The Body Mass Index in British and US Obesity Research 1960–2000’ (2014) 36 *Sociology of Health and Illness* 338.

88 Geoffrey Bowker and Susan Leigh Star, *Sorting Things Out: Classification and its Consequences* (MIT Press, 1999).

89 Eriksson and Webster (n 73) 72.

90 Gieryn (n 77) xi.

91 Ibid, 405.

92 Sheila Jasanoff, *The Fifth Branch: Science Advisors as Policymakers* (Harvard University Press, 1990) 299; see also Sheila Jasanoff, *Designs on Nature: Science and Democracy in Europe and the United States* (Princeton University Press, 2005).

93 Jasanoff, *The Fifth Branch* (n 92) 236.

94 Isabel Fletcher, ‘Obesity: A Historical Account of The Construction of a Modern Epidemic’ (PhD thesis, University of Edinburgh 2012).

95 Thomassen (n 32) 8.

96 Rosario Isasi, Erika Kleiderman and Bartha Knoppers, ‘Editing Policy to Fit the Genome?’ (2016) 351 *Science* 337.

97 Andrew Stirling, ‘Risk, Precaution and Science: Towards a More Constructive Policy Debate’ (2007) 8 *EMBO Reports* 309.

98 Christian Munthe, ‘Precautionary Principle’ in Henk ten Have (ed), *Encyclopedia of Global Bioethics* (Springer, 2015) 1–10.

99 See International Bioethics Committee, ‘Report of the IBC on Updating Its Reflection on the Human Genome and Human Rights’ (2 October 2015) 28 http://unesdoc.unesco.org/images/0023/002332/233258E.pdf (accessed 26 January 2015).

100 Ibid.

101 This section builds on a forthcoming paper, currently under review. See Agomoni Ganguli Mitra and others, ‘Reconfiguring Social Value in Health Research through the Lens of Liminality’ (forthcoming).

102 Revision of CIOMS 2002 *International Ethical Guidelines for Biomedical Research Involving Human Subjects*, ‘Guideline 1: Social Value’ http://www.cioms.ch/index.php/guideline-1 (accessed 26 January 2016).

103 Danielle Wenner, ‘The Social Value of Knowledge and International Clinical Research’ (2015) 15 *Developing World Bioethics* 76.

104 Michelle Habets, Johannes van Delden and Annelien Bredenoord, ‘The Social Value of Clinical Research’ (2014) 15 *BMC Medical Ethics* 66.

